# Efficacy of Eribulin in Soft Tissue Sarcomas

**DOI:** 10.3389/fphar.2022.869754

**Published:** 2022-03-30

**Authors:** Edward Phillips, Robin L. Jones, Paul Huang, Antonia Digklia

**Affiliations:** ^1^ Royal Marsden Hospital, London, United Kingdom; ^2^ Royal Marsden Hospital, Division of Clinical Sciences, Institute of Cancer Research, London, United Kingdom; ^3^ Division of Molecular Pathology, Institute of Cancer Research, London, United Kingdom; ^4^ Department of Oncology, Lausanne University Hospital and University of Lausanne, Lausanne, Switzerland

**Keywords:** sarcoma, eribulin and related compounds, eribulin, STS, liposarcoma, leiomyosarcoma, review

## Abstract

Soft tissue sarcomas are a highly heterogenous group of tumors with limited systemic therapy options. Eribulin, a synthetic analogue of halichondrin B, is a potent mitotic inhibitor. A phase 3 trial of previously treated advanced Liposarcoma and Leiomyosarcoma demonstrated superiority of eribulin to dacarbazine. Eribulin appears to be particularly effective for liposarcomas. It has also been shown to be a safe and effective treatment alternative to doxorubicin in patients where doxorubicin is contraindicated. From retrospective studies, eribulin has demonstrated efficacy in patients with angiosarcoma, pleomorphic sarcomas, synovial sarcomas, rhabdomyosarcomas, angiosarcomas, and myxofibrosarcomas. Future areas of development include liposomal eribulin, which may provide increased efficacy and lower toxicity, and delineation of biomarkers of response and resistance, allowing better selection of patients for treatment.

## Introduction

STS make up approximately 80% of all sarcomas. There are over 100 different subtypes (WHO Classification of Tumours Editorial Board, 2020). Liposarcoma (LPS) and Leiomyosarcoma (LMS) are two of the most common subtypes, with an annual incidence of approximately 0.9 and 0.7 per 100,000 respectively (Ducimetière et al., 2011). The mainstay of management for localized disease is complete surgical resection, with or without perioperative radiation and chemotherapy. Approximately 50% of patients with high grade tumors develop metastatic disease. The prognosis for patients with advanced disease is poor, with a median overall survival of approximately 19 months (Tap et al., 2020).

Doxorubicin, with or without ifosfamide, is the first line treatment in the majority of patients with advanced STS. There are limited second line treatments and the choice depends on STS subtype and patient performance status. Second line treatments include pazopanib, trabectedin, eribulin, and gemcitabine, with or without docetaxel or dacarbazine. Pazopanib, a tyrosine kinase inhibitor of angiogenic growth receptors, has shown superiority to placebo in a randomized placebo-controlled phase 3 trial in STS (van der Graaf et al., 2012). Trabectedin has shown superiority to dacarbazine for treatment of LPS and LMS in a phase 3 randomized clinical trial (Demetri et al., 2016). Both pazopanib and trabectedin have U.S. Food and Drug Administration (FDA) approval for the treatment of STS (National Cancer Institute, 2020). Evidence from phase 2 trials suggests efficacy of gemcitabine in STS, either alone or in combination with docetaxel, dacarbazine (Ducoulombier et al., 2016), or, more recently, nab-paclitaxel (Digklia et al., 2021). Gemcitabine does not currently have FDA approval for use in STS.

Eribulin is an inhibitor of microtubule polymerisation and is a synthetic analogue of the naturally occurring anticancer agent halichondrin B found in marine sponges (Shetty and Gupta, 2014). As well as its use in STS, it is also used in metastatic breast cancer in patients who have progressed on first and second line treatment.

In this review, we summarize preclinical and clinical data showing efficacy of eribulin in STS. We compare the efficacy across different STS subtypes. We also review potential predictive biomarkers of eribulin response as well as possible combination regimes and other future perspectives.

### Mechanism of Action of Eribulin

**FIGURE 1:**
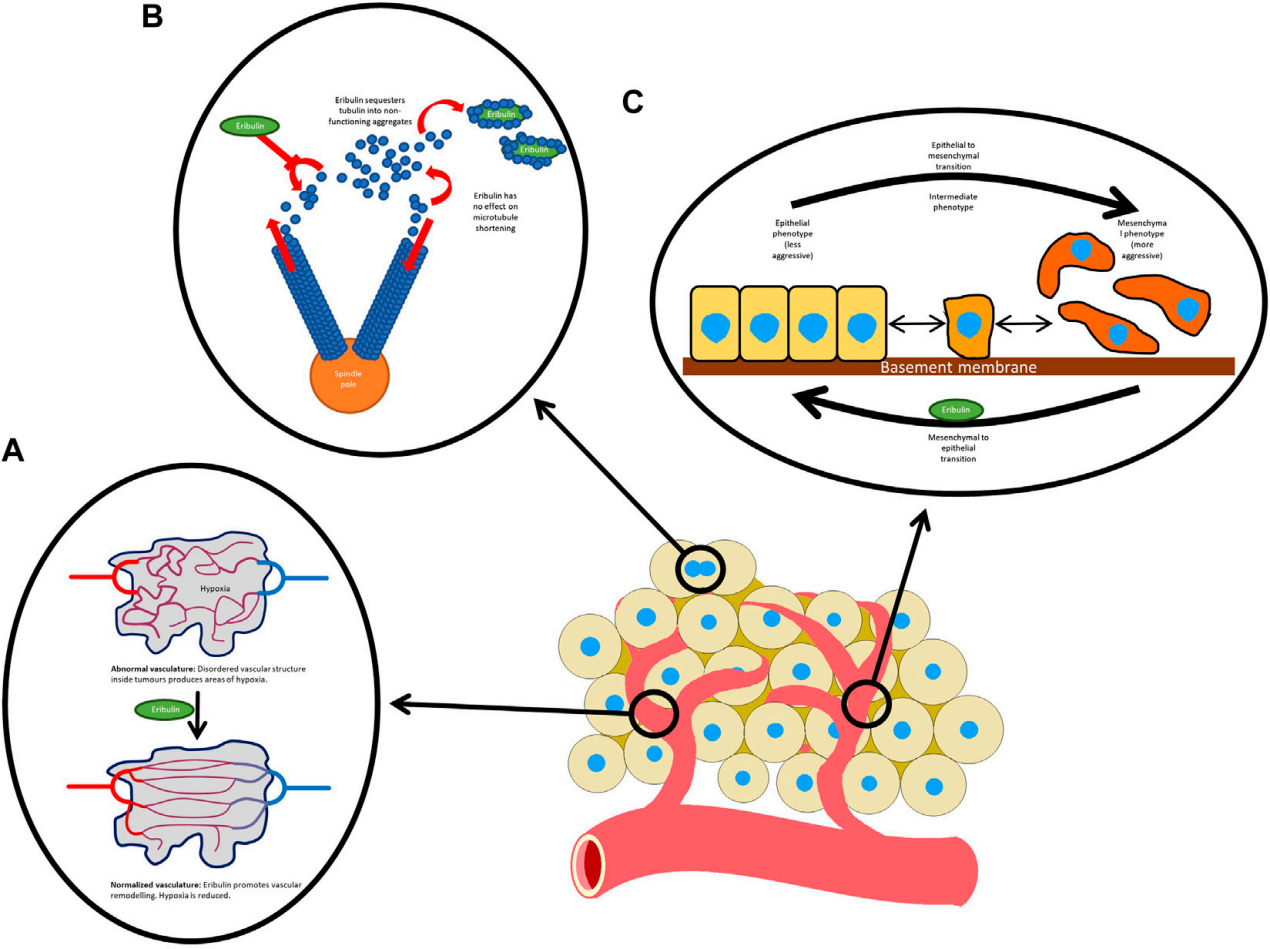
Mechanisms of action of eribulin: **(A)** normalizes the tumor vasculature; **(B)** inhibits microtubule growth without having any effect on microtubule shortening. Eribulin also sequesters tubulin, reducing the supply available to microtubules; and **(C)** reverses the mesenchymal to epithelial transition.

There are several mechanisms of action of eribulin and these are detailed in Figure 1. The predominant mechanism involves binding to the positive end of microtubules and inhibiting the growth phase (Smith et al., 2010). It displays a distinct mechanism of action from other tubulin targeting agents, including taxanes (McBride and Butler, 2012). Several other anticancer mechanisms have been suggested. In one study, vascular remodeling was demonstrated by affecting gene expression in pericytes (Agoulnik et al., 2014) and another showed improved oxygenation of tumors after treatment with eribulin (Ueda et al., 2016). Eribulin has also been shown to suppress transforming growth factor beta 1 (TGF-β1), an important growth factor that promotes cell proliferation, differentiation and metastasis (Ueda et al., 2016).

Although the predominant mechanism of action of eribulin in STS and breast cancer is likely similar, there is evidence that eribulin induces distinct differentiation patterns depending on the cell of origin. In breast cancer cells, eribulin reverses the epithelial to mesenchymal transition (Yoshida et al., 2014). Epithelial to mesenchymal transition produces a more invasive cellular phenotype and therefore is believed to underlie metastatic spread. Markers involved in the transition such as the matrix modifying enzyme MMP, the mesenchymal marker vimentin and laptm4a, a protein in volved in transport across the enodosomal and lysosomal membranes have been shown to be upregulated in STS (De Vita et al., 2017). In LPS, eribulin has been observed to promote expression of adipocytic markers and, in LMS, to promote expression of smooth muscle markers. Therefore, eribulin promotes differentiation to adipocytic and smooth muscle lineage respectively (Cortes et al., 2018).

Eribulin has also been shown to have effects on cell motility. In one study LPS cells were treated with Eribulin and compared with untreated cells. Eribulin was found to stop migratory activity in the treated cells and it was shown that Rho proteins, which are believed to be instrumental in cell migratory activity, was downregulated (De Vita et al., 2016).

### Preclinical Efficacy in Soft Tissue Sarcomas

Robust *in vitro* activity of eribulin has been shown against a wide range of STS, including fibrosarcoma, LMS, LPS and synovial sarcoma by induction of G2–M cell-cycle arrest and apoptosis (Asano et al., 2018). Interestingly, eribulin has shown improvement of vascular perfusion in LMS and clear cell sarcoma xenografts (Nakai et al., 2020). In a number of non-STS cell lines, including breast cancer and non-small cell lung cancer, combination activity of eribulin with other anticancer agents such as bevacizumab, capecitabine, carboplatin, cisplatin, doxorubicin, everolimus, gemcitabine, and palbociclib has been shown. The combination of eribulin and pazopanib has also shown a synergistic effect in myxoid, pleomorphic LPS, and LMS cell lines (Escudero et al., 2021). Combination therapies of eribulin have also shown activity in mouse xenograft models. In one study, a combination of eribulin plus an AKT inhibitor led to increased tumor suppression in a mouse xenograft STS model (Hayasaka et al., 2019). In another, combing eribulin with irinotecan resulted in tumor regression of rhabdomyosarcoma xenografts (Robles et al., 2020).

### Phase 1 Data

In a phase 1 dose finding study of 40 patients, the dose limiting toxicities of grade 3 and 4 febrile neutropenia was found at a dose of 2.0 mg/m^2^. The maximum tolerated dose was set at 1.4 mg/m^2^. No non-hematological dose limiting toxicities were seen (Morgan et al., 2015). A schedule of 1.4 mg/m^2^ of eribulin on days 1, 8, and 15 of a four weekly cycle was found to cause grade 3 or 4 neutropenia in 64% of patients (Vahdat et al., 2009). Therefore, the standard dose of eribulin is 1.4 mg/m^2^ on days 1 and 8 of a three-weekly cycle (Vahdat et al., 2009; Schöffski et al., 2011; Morgan et al., 2015). Eribulin displays linear pharmacokinetics with a rapid distribution phase followed by a slow elimination phase. Mean terminal half-life is approximately 40 h. The majority of the drug (82%) is excreted faecally (Kawai et al., 2017).

In early clinical data, eribulin has shown activity against a number of tumor types, including non-small cell lung cancer, head and neck cancer (Mukohara et al., 2012), cervical (Goel et al., 2009), urothelial and melanoma. Only one phase 1 study included a sarcoma patient. 12 patients experience stable disease, one of which was an endometrial stromal sarcoma. The mean duration of stable disease was 86 days (Tan et al., 2009).

### Non-Randomized Phase 2 Trials of Eribulin in Soft Tissue Sarcomas

In a non-randomized phase 2 trial by Schöffski et al., response to eribulin was assessed in 128 patients with STS (Schöffski et al., 2011). Eligible patients had histologically proven metastatic STS of high or intermediate grade, had received no more than one previous chemotherapeutic regime or two single chemotherapeutic drugs, and had disease progression in the last 6 months. One hundred and fifteen patients in total were assessable for the primary endpoint, which was made up of 38 LMS, 32 LPS, 19 synovial sarcomas and 26 with “other” STS. No patients with embryonal rhabdomyosarcomas, chondrosarcomas, osteosarcomas, Ewing sarcoma, gastrointestinal stromal tumors, dermatofibrosarcoma protuberans or inflammatory myofibroblastic sarcomas were included. The primary endpoint was PFS at 12 weeks.

The best results were found in the LPS group, with 15 patients (46.9%) being progression free at 12 weeks. This was followed by 12 (31.6%) in the LMS group, 4 (21.1%) in the synovial sarcoma group and 5 (19.2%) in “other” sarcomas. The five patients in the other histological subtypes were two fibroblastic sarcomas, two epithelioid sarcomas and one solitary fibrous tumor. The most common grade 3–4 adverse events (AEs) included neutropenia (52%), leukopenia (35%), anemia (7%), fatigue (7%) and raised alanine aminotransferase (5%).

Another phase 2 study, by Kawai et al., included 51 Japanese patients with STS who had received one or more prior chemotherapies for advanced disease. In that study, 16 patients had LPS, 19 had LMS and the remaining 16 consisted of synovial sarcoma, undifferentiated pleomorphic sarcoma, rhabdomyosarcoma, endometrial sarcoma, fibrosarcoma, solitary fibrous tumor, alveolar soft part sarcoma and malignant peripheral nerve sheath sarcoma. The LPS group had a median PFS of 6.8 months (95% CI 5.1–8.4) and the LMS group 2.9 months (95% CI 1.3–8.2). In the “other” sarcoma group, median PFS was 4.1 months (95% CI 2.6–5.6). The most common grade 3–4 AEs were neutropenia (86%), leukopenia (38%) and lymphopenia (33%) (Kawai et al., 2017).

### Randomized Phase 3 Trial of Eribulin Versus Dacarbazine in Previously Treated Patients With Advanced Liposarcoma or Leiomyosarcoma

To further assess the efficacy of eribulin in STS, a phase 3 randomized open label trial was undertaken (Schöffski et al., 2016). Patients with intermediate or high grade advanced LPS or LMS, who had received at least two previous systemic regimens for advanced disease and had measurable disease with RECIST 1.1, were randomized to either eribulin (1.4 mg/m^2^ on days 1 and 8) or dacarbazine (850 mg/m^2^, 1,000 mg/m^2^, or 1,200 mg/m^2^ depending on the center on day 1) in a 21-day cycle. The primary endpoint was median OS. Secondary endpoints were PFS, PFS at 12 weeks, and safety and tolerability as assessed with CTCAE v4.02. 351 patients of the 452 randomized patients were anthracycline pre-treated (77.7%). The LMS group made up 297 (67%) patients and 131 (45%) were of uterine origin.

The primary endpoint of median OS was met. The median OS was 13.5 months (95% CI 10.9–15.6) in the eribulin arm compared to 11.5 months (95% CI 9.6–13.0) in the dacarbazine arm, with a HR of 0.77 (95% CI 0.62–0.95). There was no statistically significant difference in median PFS, with 2.6 months (95% CI 1.9–2.8) PFS in both arms (95% CI 1.8–2.7). Likewise, the proportion of patients who had not progressed at 12 weeks was also similar, with 76 patients (33%, 95% CI 27.2–39.9) having not progressed after 12 weeks in the eribulin arm versus 64 patients (29%, 22.8–35.0) in the dacarbazine group. Furthermore, the response rates were low, with a non-significant difference (ORR 3.9 vs. 4.9%).

Treatment related AEs were common, with 224 (99%) and 218 (97%) patients experiencing AEs in the eribulin and dacarbazine arms respectively. Grade 3 AEs were higher in the eribulin (152, 67%) versus the dacarbazine arm (126, 56%). There was study drug withdrawal in 17 (8%) patients in the eribulin arm versus 11 (5%) in the dacarbazine arm, and dose reduction in 58 (26%) patients in the eribulin arm versus 32 (14%) in the dacarbazine arm.

### Subgroup Analysis

As a highly heterogenous group of tumors, it is unsurprising that a variety of responses would be seen in different subtypes of STS. In the preplanned OS subgroup analysis, performed in the previously described phase 3 randomized trial, LPS patients benefited from eribulin (15.6 vs. 8.4 months; HR: 0.511; 95% CI: 0.346–0.753) compared to LMS patients (12.7 vs. 13.0 months; HR: 0.927; 95% CI: 0.714–1.203). Although the small numbers involved in different LPS subtypes make it difficult to make any firm conclusions on the relative responsiveness of different LPS subtypes, the benefit from eribulin was observed across all LPS subtypes. The analysis showed more robust benefit for pleomorphic LPS, with a median OS of 22.2 months in the eribulin arm versus 6.7 months in the dacarbazine arm (HR 0.18 95% CI 0.04–0.85). The dedifferentiated subtypes had an extended median OS in the eribulin arm of 18.0 versus 9.6 months (HR 0.43 95% CI 0.23–0.79) in the dacarbazine arm. The myxoid subtype showed a more modest 13.5 months OS in the eribulin arm, versus 8.1 months in the dacarbazine arm (HR 0.79 95% CI 0.42–1.49) (Demetri et al., 2017).

Furthermore, performance status (PS) is important when selecting patients most likely to benefit from eribulin. Patients with a PS of 0 showed greater benefit when given eribulin compared to dacarbazine, with an OS of 19.9 vs. 13.1 months (HR: 0.579; 95% CI: 0.407–0.823). However, in patients with a PS of 1–2 there was no statistically significant difference in OS between the eribulin group (9.2 months) and the dacarbazine group at (9.9 months, HR: 1.09; 95% CI: 0.82–1.44).

Although there was no statistically significant benefit in the LMS cohort many patients did achieve objective responses. A retrospective analysis of archival samples of 77 LMS patients who participated in the trial were reviewed. It was found that patients with TP53 mutations were more likely, and patients with ATRX mutations less likely, to achieve disease control with eribulin. A positive correlation between TP53 mutation and PFS was shown [*p* = 0.036; HR 0.51 (95% CI 0.26–0.93)] but no impact on OS was seen. ATRX mutations were shown to have a negative impact on both PFS and OS (Wozniak et al., 2021).

Based on these data, eribulin was approved by the FDA for the treatment of unresectable or metastatic LPS in patients who had received prior anthracycline-based chemotherapy (The U.S. Food and Drug Administration, 2016) and for the treatment of inoperable STS in patients who have received previous chemotherapy for advanced or metastatic disease (Committee for Medicinal Products for Human Use and European Medicines Agency, 2016).

### Retrospective Studies in Leiomyosarcoma and Liposarcoma

After the successful phase 3 trial, there have been several retrospective real-world studies in Japanese patients who received eribulin for advanced STS. In one study, eribulin was given to 256 patients with STS of which 73 were LMS and 70 LPS (Kobayashi E. et al., 2019). Patients had received a median of two previous chemotherapy regimen prior to eribulin. It found a partial response in 5 out of 72 LMS and 2 out of 70 LPS. Eribulin has also been shown to be an effective first line treatment for STS. In six patients where doxorubicin was contraindicated due to cardiac co-morbidities or advanced age, median progression-free survival (PFS) was 9.7 months (confidence interval not reached) (Tsuchihashi et al., 2020). In a recent retrospective study of 23 patients with advanced STS (predominantly LPS and LMS), body composition has been shown to be a predictor of eribulin toxicity. Grade 4 hematological toxicities were significantly higher in those with low skeletal muscle gauge (*p* = 0.02). Grade 3 and 4 non-hematological toxicities were also associated with low skeletal muscle gauge (*p* = 0.04) as well as low serum albumin level (*p* = 0.02) (Kobayashi H. et al., 2019).

### Angiosarcomas

Angiosarcomas are a highly aggressive tumor of endothelial tissue. They represent 1–2% of all sarcomas. They can develop throughout the body but about 60% are cutaneous (Cao et al., 2019). Anthracyclines, such as doxorubicin, and taxanes can be used to treat advanced angiosarcomas. Taxanes are usually preferred in older patients with more comorbidities (Fujisawa et al., 2020).

In a single-arm prospective observational study of 25 patients who had previous treatment with a taxane, eribulin was given for cutaneous angiosarcoma (Fujisawa et al., 2020). The age of enrolled patients ranged from 62 to 88 (median age 74) years, and 88% had an Eastern Cooperative Oncology Group (ECOG) performance status of 0 or 1. Median OS was 8.6 months and PFS 3.0 months. The best overall response rate (ORR) was 20% (5 out of 25). A total of 16 grade 3 or 4 Serious Adverse Events (SAEs) were seen. 56% (14 out of 25) underwent dose reductions and 44% (11 out of 25) had their treatments postponed due to AEs. Excellent responses to eribulin in scalp cutaneous angiosarcomas have also been reported in two cases reports. In the first case, a patient with previous scalp angiosarcoma presented with lung metastases. The patient was treated with eribulin and his disease remained well controlled after nine cycles of treatment (Wada et al., 2018). In the second case, a very good partial response was seen in a local recurrence of a scalp angiosarcoma when treated with eribulin (Iwamoto et al., 2018). In another case report, eribulin was given as an eighth line treatment for metastatic cardiac angiosarcoma with a partial response maintained for 4 months (Inagaki et al., 2018).

### Retrospective Studies in Other Subtypes

There are many other subtypes of STS that have limited treatment options. Small numbers of non LPS and LMS STS subtypes were included in phase 1 and 2 studies. These data are summarized in, Table 2. In a real-world observational study of 256 Japanese patients, eribulin was shown to have antitumor activity against multiple subtypes (Kobayashi E. et al., 2019).

**TABLE 1:** Phase 2 and 3 clinical trials for eribulin in STS.

Author	Study type	ECOG Performance Status	Soft tissue sarcoma subtype	Number of patients receiving eribulin	Median overall survival (months)	Median progression free survival (months)
Schöffski et al. (2016)	Randomised phase 3 trial versus dacarbazine	0 (49%)	LPS and LMS	228	15.6	2.6
		1 (50%)				
		2 (1%)				
Schöffski et al. (2011)	Non-randomised, single arm phase 2 trial	0 (64%)	LPS	32	Not reported	2.6
		1 (36%)	LMS	38	Not reported	2.9
			Synovial	19	Not reported	2.6
			Other sarcoma	26	Not reported	2.1
Kawai et al. (2017)	Non-randomised, single arm phase 2 trial	0 (53%)	LPS and LMS	35	17.0	5.5
		1 (47%)	Other sarcoma	16	7.6	2.0

**TABLE 2:** Phase 1, 2, and 3 data for non LPS and non LMS STS subtypes.

Author	Phase	Total number of non LPS or LMS STS	STS subtypes with stable disease or better
Tan et al. (2009)	1	1	Endometrial stromal sarcoma
Schöffski et al. (2011)	2	45	4 synovial sarcomas
			2 fibroblastic sarcomas
			2 epithelioid sarcomas
			1 solitary fibrous tumor
Kawai et al. (2017)	2	16	Endometrial stromal sarcoma (2/2 patients)
			Synovial sarcoma (1/3 patients)
			Solitary fibrous tumor (1/2 patients)
			Fibrosarcoma (1/2 patients)
Schöffski et al. (2016)	3	0	

Median age in the study was 62 (range 17–87) years and 84% (214 out of 256) had an ECOG performance status of 0 or 1. Median time from diagnosis to initiation of eribulin was 2.5 years (range 0.2–29.2). Target lesions were most commonly retroperitoneal or intraperitoneal (40.4%). The most common number of prior chemotherapy regimens was one (31.8%) followed by two (29.0%). Only 7.1% received eribulin first line. The most common prior chemotherapeutic regimen was doxorubicin monotherapy (36.9%), followed by pazopanib (32.2%), gemcitabine and docetaxel (26.7%) and doxorubicin and ifosfamide (22.7%). A total of 174 grade 3 or 4 SAEs were seen. The most AE was neutropenia, which was seen in 52.5%. Fifty-five patients (21.6%) underwent dose reduction.

A partial response was seen in 17 patients. Excluding LPS and LMS, 10 out of 143 had partial responses. This was seen in 2 out of 19 undifferentiated pleomorphic sarcomas, 3 out of 15 synovial sarcomas and 2 out of 12 rhabdomyosarcomas. A partial response was also seen in one patient, each in the angiosarcomas (14 patients in total), myxofibrosarcoma (5 patients in total) and undifferentiated round cell sarcoma (1 patient in total).

Another retrospective Japanese study of 82 STS patients treated with eribulin included 45 patients that had neither LPS nor LMS STS. Overall, 72% had received prior anthracycline based chemotherapy and 75% had a PS of 0 or 1. The 45 patients that had neither LPS nor LMS STS consisted of 13 undifferentiated pleomorphic sarcomas, six synovial sarcomas, five malignant peripheral nerve sheath tumors and 21 unspecified subtypes. A partial response was seen in one myxofibrosarcoma. Stable disease for at least 6 months was seen in one undifferentiated pleomorphic sarcoma, one synovial sarcoma and one sclerosing epithelioid fibrosarcoma (Nakamura et al., 2019). Eribulin has shown a clinically meaningful level of activity in several STS subtypes in the Japanese population. This has led to approval in Japan of eribulin for all pre-treated STS patients in 2016. (Eisai Global, 2016).

## Future Perspectives

Liposomal preparations of several chemotherapeutic agents have been developed and have the advantage of improved targeting of tumor sites and decreased toxicity. This has been shown to produce improved efficacy in several cases (Fanciullino and Ciccolini, 2009). A liposomal formulation of eribulin has been developed, which aims to replicate some of these successes. In pre-clinical studies, changes to the liposome formulation reduced the release rate of the liposome, reducing Cmax and increasing the half-life (Yu et al., 2013). This could allow higher doses to be used while reducing associated toxicities. A phase 1 study, which did not include any patients with STS, has shown a good side effect profile and response rates that compared favorably to non-liposomal eribulin (Evans et al., 2019). Pending results from further trials in other tumor types, liposomal eribulin is a promising future therapy for STS.

Patient selection is key in determining response to eribulin in STS and identification of biomarker signatures is key (Emambux and Italiano, 2017). In one study of 52 patients with triple negative breast cancer, lack of the transcription co-repressor transducin-like enhancer of split 3 (TLE3) was associated with poorer outcomes when treated with eribulin (Kashiwagi et al., 2017a). In another study, mutations in the Phosphoinositide 3-kinase and AKT pathway in HER-2 negative breast cancer xenografts was also linked to a poorer response to eribulin (Gris-Oliver et al., 2021). In osteosarcomas, increased expression of the drug efflux pump P-glycoprotein and the tubulin isotype βIII-tubulin was associated with lower responsiveness to eribulin (Sampson et al., 2016). It is unclear whether similar mechanisms are involved in STS or whether other markers of response and resistance are important. Future drug targets, such as P-glycoprotein, may increase the efficacy of eribulin.

Correlation of microRNA expression levels with oncological outcomes in various cancer types has also been investigated. A panel of a total 26 miRNAs that correlate with eribulin response (*p* < 0.05) have been identified by using archival tumor tissue from patients treated in the non-randomized phase 2 trial of eribulin. However, this hypothesis should be validated by prospective trials (Wiemer et al., 2017).

Eribulin has been shown to have important effects on the tumor immune microenvironment. In one study in breast cancer, patients with higher levels of tumor infiltrating lymphocytes (TILs) receiving eribulin had a better disease-free survival than those with lower levels of TILs (Kashiwagi et al., 2017b). The epithelial to mesenchymal transition is believed to be detrimental to the immune microenvironment. Eribulin has been shown to reverse this process (De Vita et al., 2017). Therefore, reversal of this may promote TIL cytotoxic activity. In a retrospective cohort study in breast cancer, tissue samples were obtained before and after treatment in ten patients. Five patients were deemed responders and five non-responders. PD-L1 expression became negative in six patients. This was significantly associated with response to eribulin (*p* = 0.024) (Goto et al., 2018). A recent phase I/II trial of eribulin in combination with pembrolizumab showed promising antitumor activity in metastatic triple negative breast cancer. In the subgroup analysis both PD-L1 positivity and being treated in the first line setting was associated with a greater ORR (Tolaney et al., 2021). This suggests immunotherapies may have a synergistic effect in combination with eribulin. However, a phase 2 trial of 19 LMS patients treated with eribulin in combination with pembrolizumab found the PFS at 12 weeks to be only 42.1%. This failed to reach the primary endpoint of a 60% PFS at 12 weeks (Nathenson et al., 2020). Recently, data from the LPS cohort was presented showing a PFS rate at 12 weeks of 67% and a median PFS of 27 weeks (Nathenson et al., 2021). Furthermore, the authors reported that three patients with angiosarcoma showed significant responses in addition to one patient with SMARCA4 deficient thoracic sarcoma.

Eribulin in combination with other anticancer therapies may produce synergistic anticancer activity. Cyclin dependent kinase (CDK) 4/6 inhibitors restrict phosphorylation of the retinoblastoma protein stopping cells from exiting G1 and proceeding through the cell cycle. They have found widespread use in advanced hormone receptor positive breast cancer. A phase 2 trial of palbociclib in well differentiated or dedifferentiated LPS showed a favorable PFS of 17.9 weeks (Dickson et al., 2016). A combination schedule of CDK 4/6 inhibitors with eribulin may have a synergistic effect due to their distinct actions on cell division. The ERIGE trial was a phase 2 trial of eribulin in combination with gemcitabine for advanced triple negative breast cancer. This found an overall response rate of 37.3%. In a recent proof of concept phase 2 trial, the combination of eribulin with gemcitabine has shown encouraging results in advanced liposarcoma and leiomyosarcoma pretreated patients with a 3 months PFS rate of 73% (Kim et al., 2021). In another study, the combination of eribulin and the AKT inhibitor MK-2206 was associated with synergistic activity in both sarcoma cell lines and in STS murine xenograft mouse models (Hayasaka et al., 2019). The combination of lenvatinib, a multiple kinase inhibitor with anti-angiogenic activity, and eribulin may also show synergistic anticancer activity. In a single arm phase 1b/II study of lenvatinib and eribulin in 14 LMS and 6 LPS the overall response rate by RECIST 1.1 was found to be 27% (5/18). 15 patients experienced at least one grade 3 or 4 AE with hypertension (4 patients, 27%), hand-foot-syndrome (4 patients, 27%) and proteinuria (3 patients, 20%) being the most common. These studies suggest possible roles of CDK4/6 inhibitors, gemcitabine, AKT inhibitors and lenvatinib as combination therapies with eribulin.

Retroperitoneal sarcomas (RPS) make up about 16% of all sarcomas (Carbone et al., 2021). Local recurrence is more common post-resection than at other sites and a large subset of patients have unresectable disease at diagnosis. The use of neoadjuvant chemotherapies would aim to shrink RPS and allow successful resection of previously unresectable tumors, and to improve margin status thereby reducing the chance of recurrence. Ifosfomide and doxorubicin can be given for RPS as a neoadjuvant therapy. However, due to poor response rates and high levels of toxicities, they are not always suitable (Almond et al., 2018). As eribulin has shown efficacy in metastatic LPS, it may be an appropriate neoadjuvant therapy in locally advanced retroperitoneal LPS. An ongoing phase 1b clinical trial is using neoadjuvant eribulin and radiotherapy in RPS. The primary endpoint is determination of the recommended phase 2 dose. Secondary endpoints include the assessment of anti-tumor activity of combined eribulin and radiotherapy, and surgical outcomes of retroperitoneal LPS after neoadjuvant chemoradiation. Estimated completion date of this trial is February 2022 (U. S. National Library of Medicine, 2021).

It is still unclear why eribulin is more effective at extending OS than PFS. To date, this was replicated with similar results in the EMBRACE trial, a phase 3 study of eribulin in advanced breast cancer (Cortes et al., 2011). One possible explanation is that eribulin sensitizes tumor cells to later lines of chemotherapy. In one pre-clinical study, eribulin promoted vascular remodeling in tumors and improved perfusion to tumor cells. This was shown to improve the anti-tumor activity of capecitabine (Funahashi et al., 2014). Another explanation is that eribulin may promote immune system mediated anticancer activity which may continue after eribulin has been stopped.

## Conclusion

Eribulin is licensed by the FDA for the treatment of unresectable and metastatic liposarcoma for patients who have received prior chemotherapy with an anthracycline. It is also useful off-label as a first line treatment, particularly in patients at risk of doxorubicin toxicity. Responses have also been demonstrated in LMS, however it failed to show superiority to dacarbazine in a phase 3 trial so any use in LMS would be off-label. Responses have also been demonstrated in angiosarcomas, undifferentiated pleomorphic sarcomas, synovial sarcomas and rhabdomyosarcomas. Low numbers of patients in these cohorts make comparisons with other chemotherapeutic regimes difficult.

In the future, biomarkers such as P-glycoprotein and miRNAs may improve patient selection. The development of a liposomal formulation of eribulin may allow for higher doses to reach tumor cells while reducing the side effect profile. Clinical trials for this in breast cancer are ongoing. Eribulin may have synergistic effects when combined with other therapies such as CDK 4/6 inhibitors, AKT inhibitors and immunotherapies. There may be a role for eribulin as a neoadjuvant treatment for RPS and a clinical trial is ongoing. It is uncertain why eribulin extends OS but not PFS. However, the most likely explanation is that eribulin sensitizes tumor cells to later lines of chemotherapy.
